# Challenges and Feasibility of Co-Design Methods for Improving Parent Information in Maternity Care

**DOI:** 10.3390/ijerph19073764

**Published:** 2022-03-22

**Authors:** Kathryn Kynoch, Anthony Tuckett, Annie McArdle, Mary-Anne Ramis

**Affiliations:** 1 Mater Health, Mater Misericordiae Limited, Newstead, QLD 4006, Australia; kathryn.kynoch@mater.org.au (K.K.); annie.harris@hotmail.com (A.M.); 2 Queensland Centre for Evidence Based Nursing and Midwifery: A Joanna Briggs Centre of Excellence, Mater Health, Newstead, QLD 4006, Australia; 3 Australian Centre for Health Services Innovation (AusHSI), School of Public Health and Social Work, Faculty of Health, Queensland University of Technology, Brisbane, QLD 4000, Australia; 4 Curtin School of Nursing, Curtin University, Perth, WA 6102, Australia; anthony.tuckett@curtin.edu.au

**Keywords:** co-design, experience-based co-design, EBCD, postnatal, information needs, participatory action research, service delivery

## Abstract

This study explored the feasibility of using experience-based co-design methods (EBCD), based on participatory action principles, to improve service delivery regarding parent information needs within a metropolitan postnatal maternity unit. Data were collected from January 2018 to March 2019 from parents and staff using surveys, video interviews, a focus group and ward observations of episodes where parents were provided information. Participants included postnatal mothers who had recently given birth, their partners and hospital staff. Survey results (*n* = 31) were positive regarding content and satisfaction with information delivery. Data from the staff focus group (seven participants) and in-depth video interviews with mothers (*n* = 4) identified common themes, including challenges to information delivery due to time pressures, the value of breastfeeding advice and environmental influences. Overall, parents were satisfied with the information delivered; however, inconsistencies were present, with time pressures and other environmental factors reported as influencing the process. Staff and parents both identified the amount of content being delivered in such a short time frame as a major challenge and tailoring information was difficult due to individual experiences and circumstances. Additional resources or alternative methods are suggested for conducting future studies to capture patient experience within a similar busy hospital setting.

## 1. Introduction

The early post-natal period is a demanding time for new parents. Once mother and baby are physiologically stable, midwifery staff play an integral role in providing parents with enough information to safely care for their newborn baby after discharge [1]. Midwives, nurses and doctors all provide information to parents within maternity settings; however, the significance of midwifery education and information has been noted in the literature. Providing consistent information is associated with parents feeling more secure in their role as they transition to home [2]. Information provision has also been linked to overall patient satisfaction, with mothers who felt they had not received sufficient and/or appropriate information reporting to be dissatisfied with their entire hospital stay [1,3]. Early hospital discharge places further challenges on parental preparation.

There is no accepted definition on early discharge timeframes, with average length of stay (LOS) ranging from less than 12 h following vaginal delivery to between 2 and 4 days following caesarean section [4,5,6]. These time frames may be influenced by maternal comorbidity or organizational factors [7]. From a service perspective, early discharge is proposed to save money; however, risk of readmission for mother and baby exists, which may negate cost savings. Potential benefits of early discharge include increased family togetherness; greater rest and sleep within the home environment; decreased risk of exposure to nosocomial infections; enhanced confidence in caring for the baby in the home environment; and fewer breastfeeding problems due to limited conflicting advice and decreased exposure of the infant to hospital schedules [5]. Opponents suggest early discharge can lead to serious knowledge deficits if women are overwhelmed with large volumes of information at a time when they are not ready to receive it, potentially resulting in negative outcomes [8]. Studies of maternal cognition in the postpartum period report cognitive deficits, especially within the first 24 h [8], with verbal memory and word recall proposed to be significantly reduced [9]. Information overload in such a short time, alongside a decreased ability to process information, has resulted in growing interest about how midwives and other health care professionals can better meet parent information needs.

Breastfeeding support is a key focus of education and information that midwives provide while in the immediate postnatal period [10]. Swerts et al. [10] conducted a systematic review of 11 qualitative studies, which identified the time pressures faced by midwives to deliver information and provide breastfeeding support in the hospital context. The findings highlight the impact of interruptions, not meeting mother’s expectations and the technical aspect of breastfeeding support, which often overrides the mother’s desire for a ‘companion-like’ relationship [10]. Paradoxically, the review also identified the role of pediatricians as providers of breastfeeding information, highlighting the challenge in determining where and how parents wish to receive information.

The most effective way to provide information in the early postnatal period to promote parental confidence and infant-parental bonding is unclear [11]. Traditional methods, such as face-to-face education provided by a midwife, with supporting written information [12], are being augmented or replaced with increasing use of technology and/or social media [1,5,13]. To effectively support new parents’ information needs, it is necessary to identify what information is being provided, what additional information parents need or want, and their preferred delivery methods. The aim of this project was to examine the feasibility of conducting an information needs co-design study in a busy maternity setting. Obtaining perspectives from midwives and other health professionals within the maternity setting may also help to uncover barriers or facilitators to information transfer prior to discharge.

## 2. Materials and Methods

### 2.1. Design

Experience-based co-design methods outlined by Robert and colleagues [14] were used as a guide for this study. Experience-based co-design (EBCD) enables staff and patients (and other stakeholders) to work together, to improve care [14,15]. The methodology comprises video stories from patients in addition to ethnographic observations of routine care, to capture different perspectives. Patients and staff are then encouraged to work together as equal partners to co-design and improve processes and services [16]. Ideally, EBCD focuses on patient and staff experiences rather than attitudes or opinions, with qualitative data adding rich insights into patient experience. Aligning to participatory action research methods, a growing body of evidence demonstrates effectiveness and support of the approach, particularly within local contexts [15,17]. A systematic review of 20 studies using EBCD methods [16] identified challenges in conducting such studies and highlighted inconsistency in reporting of results.

### 2.2. Setting

The study took place in a tertiary maternity hospital in Brisbane, Australia. The hospital provides maternity and neonatal care services, with over 10,000 births per year. One postnatal ward with a capacity of 41 beds was the focus for this study. The average length of stay in 2018 was 2.13 days for uncomplicated vaginal delivery and 3.08 days for uncomplicated caesarean section.

### 2.3. Participants

Participants included postnatal mothers, their partners and hospital staff. Mothers and partners were required to be over the age of 18, understand English sufficiently to participate (or had a family member to assist) and had recently given birth at the hospital. There were no limits on parity nor prior admissions. Mothers who had a baby in the Neonatal Critical Care Unit or had a stillbirth/neonatal death were not approached.

The study population for staff included midwives, obstetric medical staff (including neonatal specialists), physiotherapists, occupational therapists and social workers. Two weeks before project commencement, project flyers and information posters were placed around the postnatal unit to advise staff and parents of the project. Staff were also informed of the study during in-service sessions within this same period.

### 2.4. Data Collection Methods

#### 2.4.1. Survey Methods

After ethical and hospital governance approvals were obtained (see Section 2.5), data were collected from parents via paper surveys. Two of the research team members (a research midwife who did not work on the floor and a research nurse) attended morning briefings on the ward each day for two weeks. With consent of the Nurse Unit Manager, they identified parents who were being discharged and who met the inclusion criteria. These parents were approached by the research team members and given a project information leaflet. They were then asked if they wished to complete the survey. If agreeable, the survey was left with the parents, who were asked to deposit the completed forms into a central and secure location on the ward as they left the unit. Completion of the survey was seen as consent to participate. Parents were also asked if they wished to participate in a video interview at a time convenient to them after discharge. Parents who were interested were provided another information sheet with contact details of the research team, and were offered the choice of contacting the researcher or providing their email address to the research team members for follow up. Section 2.4.4 explains this data collection process further.

#### 2.4.2. Survey Instrument

The Satisfaction with Postnatal Discharge Information questionnaire comprised nine questions on satisfaction with information and education provided during their postnatal stay in hospital and at discharge. As the questions pertained to our specific setting, the survey had not been validated in other contexts. A five-point Likert scale was used for parents to rate their response from strongly disagree to strongly agree. Partners or mothers were able to answer the survey, with the only demographic detail collected being how many children the parent had (e.g., first baby, second etc.). No identifiable details were captured in the survey as the goal was to identify perspectives on satisfaction only. The survey questions are included as Appendix A.

#### 2.4.3. Ward Observation Data Collection

Observational data were obtained by members of the research team, with the view to identify information exchange between a staff member and parent at various times. Prior to collecting any data, consent was obtained from the unit manager, and staff members who were working on the floor were provided project information and asked to sign consent forms prior to any observations taking place. Time for questions was also provided. Parents were not asked for written consent for this stage of data collection due to the desire not to interrupt daily workflow processes and to collect data from the naturalistic setting, as is common with this method [18]. However, the midwives were asked to inform parents that the observations were occurring and that they could request not to be observed. An observation record sheet was developed and used by the researchers, and field notes were taken; however, no identifiable staff or parent information was collected. An example of the data collection form is included as Appendix B. Ward observations were carried out across four sessions: two information sessions on baby care, one session on discharge information specific to maternal care postpartum and one group breastfeeding education class conducted by a midwife/lactation consultant.

#### 2.4.4. Interview Data Collection

Parents who agreed to the follow-up were emailed 2–4 weeks after discharge to see if they wished to participate in further data collection via individual, semi-structured video-taped interviews. Participants who agreed came back to the hospital for the interviews, which were videotaped in a private room within the hospital at a time convenient to the mother. A research midwife, not involved with the participants’ postnatal care, asked broad questions about their postnatal hospital experience with focus on the information received. At completion of the interview, participants were provided a car parking voucher and a small token gift.

#### 2.4.5. Focus Group Data Collection

Data from health professionals were collected via a one-off focus group, conducted in a private room in the hospital, away from the postnatal unit. The focus group was open to any health professional who worked on the postnatal ward; this included obstetricians, neonatal doctors, physiotherapists, social workers, medical resident doctors and pediatricians. The moderator for the focus group was a research midwife, who was also a member of the research team. The focus group was audiotaped, and the recording was transcribed by another research team member, who was also present during the discussion.

### 2.5. Data Analysis and Managemnt

Survey and categorical data were summarized using frequencies and percentages. We report any missing data but, due to the type of improvement project, it was not anticipated that missing data would impact the overall study aims. Qualitative data from free text responses and transcripts were analyzed for repeated themes and subjects, and pooled into thematic and topical categories.

The results from the hardcopy surveys were entered into an Excel file by a member of the research team. Completed surveys and transcripts had no identifying information attached. All electronic data including video and transcript files were stored on a secure hard drive, which was password protected and only accessible by the researchers.

### 2.6. Ethical Considerations

Ethical and Governance approvals were obtained from the Hospital Human Research Ethics committee (Ref No. HREC17/MHS/40), prior to any data collection. All parents were provided participant information consent forms for the survey, and individual interviews and written consent was obtained from parents and staff for the individual interviews and focus group respectively.

## 3. Results

### 3.1. Survey Results

A total of 31 surveys were received, of which, a majority were completed by the mother (86.7%; *n* = 26). Just under half of the sample were first time parents (48.4%; *n* = 15). Most of the respondents agreed (16.1%; *n* = 5) or strongly agreed (67.7%; *n* = 21) that the midwives understood their information needs. Overall, 61.3% (*n* = 27) of parents were satisfied with their discharge information; however, 9.7% (*n* = 3) were neither satisfied nor dissatisfied, and one respondent strongly disagreed with this item. Further results for the survey are seen in Table 1.

### 3.2. Ward Observations

In total, two hours and 20 min of information provision were observed. Partners were present for three of the four sessions. The content delivered during these sessions is presented in Table 2.

Observational data identified written materials were delivered in 75% of cases; however, in half of these situations, the written materials were observed as being placed somewhere by the mother (e.g., in a drawer), which was unclear to the observer. Subsequently, it was not clear if the materials were read prior to leaving hospital or if these materials were taken home with the parent/s. Staff did ask the parents if they had any questions in three out of four interactions (75%) but spontaneous questions were asked by the parents in only half (50%) of the sessions.

### 3.3. Individual Interviews

From 13 women approached, four individual interviews (average time = 28 min) were conducted and videotaped. Each of the interviewees were first time parents and, despite attending antenatal classes, all the mothers said that they felt unprepared for being a parent and at times were overwhelmed with the amount of information received on different topics within such a short timeframe.

“*…You think about the pregnancy and all the challenges that come with that and then the birth which is obviously a huge worry and kind of where your anxieties are focused and I think for me I was just concentrating on getting past that point and thinking, the rest will come…so I wasn’t really reading a lot about how to feed, the signs you have to look out for… I didn’t really know much at all.*”

(P1)

#### 3.3.1. Theme 1: Conflicting Advice

Conflicting advice exacerbated feelings of being overwhelmed and participants reported that they would refrain from asking questions at times, due to the varying responses received by different members of the health care team. As reported by one participant:

“*…different information from different midwives, umm, can be quite overwhelming…and you don’t want to kind of say…the other midwife told me to… you just kind of sit there quietly because you don’t want to get anyone in trouble…so then you are conflicted in that information… I don’t think it’s just in the hospital, I think it’s everywhere…but in the first couple of days when you’ve got a newborn it can be very overwhelming… to have that different information is all very overwhelming.*”

(P1)

Another mother reported on conflicting advice from different health professionals regarding recovery after a caesarean section. She stated:

“*…so, I had no idea, should I be resting, or up and walking. I had no idea what to do and nobody agreed on what I should do so I was just like, don’t ask any questions.*”

(P3)

Generally, participants seemed to agree they had received information on a variety of topics such as settling techniques, baby care and establishing breastfeeding; however, some mothers felt specific information was missed during their stay and would have been helpful prior to discharge, such as being shown how to bath their baby, cord care, monitoring baby output, wound care (maternal) and when to seek help. The difficulty in being able to deliver tailored information was highlighted by various and contradictory responses on these topics from the mothers themselves. For example, one mother requested a written document to support her breastfeeding knowledge, whereas another mother who did not breastfeed did not want any extra information. One mother was happy with information received about cord care, whereas another stated they had not been told anything and expected that someone would remove the baby’s cord while they were in hospital. Information on mental health was requested to be more specific by one mother to differentiate between the ‘baby blue’s’ and postnatal depression and/or normal and abnormal levels of anxiety. Although some participants received general information on where and when to contact health professionals if needed (e.g., GP, hospital, parent support unit, ED) specific written information on this content was also desired by others. As one mother stated:

“*We were given information, but I feel it was almost deliberately non-specific.*”

(P4)

#### 3.3.2. Theme 2: Environment Influences

The busy environment was a factor reported on by all the participants and, although they understood that was the nature of the unit, it impacted on the information received and their personal experience. As reported by one participant:

“*It’s a crazy time and you have so much information coming at you, I couldn’t really remember who told me what…*”

(P2)

This was supported by another mother who was happy with her care, recounting the different personnel she saw in one day:

“*…Nurse Midwives, Lactation consultants (twice), Pediatrician, Physio, the Registrar, baby hearing person…it was amazing…*”

(P4)

The number of staff attending to the women was identified as impacting on information transfer in different ways. One participant reported on repetition:

“*…a lot of different personnel asking the same questions.*”

(P2)

In contrast, another participant articulated confusion around the specific roles of the different staff attending to her. She reported:

“*I wasn’t entirely clear … I saw a lot of different doctors, which wasn’t really a problem, they were all lovely and friendly, but I did see maybe three doctors on the second day, and I wasn’t entirely clear how they related to the previous doctor…it was a little confusing to see new faces all the time…*”

(P1)

The overall busy-ness of the unit impacted the discharge experience for the interviewees. Three of the four women reported they would have liked to have someone to say good-bye and thank-you to, at the time of discharge. As stated by one mother:

“*…When we were leaving there was no one to say goodbye to. I think that was important for us, we wanted to say thank you for your help and goodbye because it felt like such a big thing taking our baby home…we realized it was a very busy morning …I felt so grateful for everything, and it would have been nice to just have a hug and say thank you so much… I felt we weren’t parents before we came in, but we were parents then [on discharge from hospital]. It’s a really huge transition and you just want a face to thank.*”

(P1)

#### 3.3.3. Theme 3: Breastfeeding Information

Information received at the breastfeeding clinic was described by most participants as very helpful, despite the fast-paced nature of the ward. This was summarized by one mother as:

“*…they certainly rushed it, things were discussed quickly, and you got your 30 s with a lactation consultant, but it’s not for their want of trying, they were so good, they were so professional and so kind and so patient… but there were 12 of us…and we only had 40 min…*”

(P4)

Again, however, there were contradictory responses. One mother found the information on feeding overwhelming:

“*…so, I just felt like, completely overwhelmed with so much information about breastfeeding techniques…*”

(P3)

Information on how to sustain breastfeeding was requested, and potential challenges that may eventuate, along with strategies to manage issues such as low or over-supply or attachment issues. Conversely, two mothers said that although they understood the need to promote breastfeeding within the hospital, some information about formula feeding would be appreciated.

Commonly, the mothers all reported being satisfied with their midwifery care and support given throughout their stay.

“*That time we had in hospital was exceptional, it was really good...All the information we got was very good*”

(P4)

### 3.4. Health Professional Focus Group

The final data collection method utilized a focus group of seven (7) health professionals representing obstetric and neonatal medicine, allied health (physiotherapy and social work), midwives and midwifery managers. The interview lasted just under an hour (57 min) and after the discussion was transcribed, it was returned to some group members for confirmation. The transcription was reviewed by research staff several times to look for re-occurring themes.

Each professional provided information that was pertinent to their specialty, but they all agreed that two main barriers to providing information were time pressures and the number of staff involved in care. As one participant reported:

“*I’d be really interested to count up a time in motion study of between the women hitting the postnatal floor and leaving. You know, say she has a 24 h admission for a vaginal birth, it’s likely to be 18 h of activity that needs to happen to that woman if someone counted up [group agreement], God knows when she’s going to feed the baby, you think about the obs [observations] on the baby, the obs on the mother, all the education, the classes, the physio, the pharmacist…that’s just a huge amount of stuff that’s supposed to happen in this tiny amount of time when you’re [the mother] not at your best.*”

This was reinforced by another participant who acknowledged that, even though copious information was being delivered, mothers were not always ready to receive the information. They stated:

“*They’re [mothers] not sometimes in a good space to take on that information and you know they’re not hearing anything you’re talking about because they’re in pain, or they might come to a class and you’re just about to start watching a feed and they’ve got to go and have their hearing screening or the midwifes coming in saying they need a jaundice test…*”

Empathy was expressed for the midwives working on the ward:

“*They’re under the pump and they’re so pressured and they’re really busy on that ward that perhaps some of these things get missed occasionally.*”

Another staff member expressed the following:

“*I think the staff there give their best you know with the time constraints that they have. It’s unfortunate, a lot of them would like to give more but they’re certainly strapped for time, but they’re passionate…*”

Discussion continued regarding discharge information to prepare parents for parenting after leaving hospital. There was mention of brochures being handed out, physiotherapy treatment for physiological return to function and efforts to address mothers’ needs regarding wound, back and pelvic floor care. Other staff discussed the importance of providing information to mothers on symptoms of post-natal depression. Staff stated that they provided information to mothers regarding signs and symptoms for both mother and baby that might require medical review after discharge. The breastfeeding clinic was seen universally as a positive education session, as one of the doctors stated:

“*Talking to the mums, you can tell the ones who have been to the breastfeeding clinic. Their dialogue, the way they talk back to you is like, ok, you know what’s going on…*”

English not being a first language was discussed as a challenge to information delivery and, although interpreters were frequently used, it was acknowledged across the group that having an on-site interpreter was invaluable, especially for emergency situations. Despite having white-boards for communication in the patients’ bed area, it was noted that many mothers were unsure when they were being discharged to home. A desire for easily accessible postnatal information was discussed among the group, with focus on the utility of a mobile phone application.

## 4. Discussion

The aim of this study was to apply co-design methods to examine parent and staff perspectives of information provision in a busy maternity setting. Following EBCD principles [14,15], we undertook several methods of data collection to achieve this aim. This study provides important insights into the feasibility of using co-design methods in this type of setting because, despite rigorous planning procedures, our paper identified challenges in recruitment and follow-up of participants. Despite this, our results demonstrate the impact that midwives have on providing information to parents to prepare them for caring for their baby.

Although the survey results were positive relating to parent’s satisfaction with information delivery, a clear message was received from both parent and staff perspectives regarding time pressures faced within the unit and how these impacted on the experience. Early discharge after birth is a common practice globally and, although evidence suggests it is a safe practice for mothers of term babies [4,5], it has been noted that the quality of studies and the ambiguity in defining a time frame of early discharge limits certainty of this evidence [4]. Regardless, it has been reported that mothers who are discharged early following vaginal delivery may seek advice after discharge on topics such as breast-feeding and newborn feeding, baby hygiene and umbilical care, in addition to information on how to care for themselves postnatally [4]. There is currently limited evidence on how best to support parents with information for these needs once discharged from hospital, following vaginal or caesarean section delivery. Our study suggests a need for individualized and multimodal parent information delivery that meets the needs of parents and staff in delivering consistent and unified messages. However, despite the importance of providing such tailored information [19], we also identified some challenges in meeting individualized needs due to the personalized experience of each mother and family unit. Further studies with larger and more diverse samples are necessary to investigate the most effective strategies for this context.

The breastfeeding clinic was a positive educational experience in our context and highlights the impact of being supported by not only the midwife/lactation consultant in this setting, but also by peers. Being surrounded by others going through a similar experience can be a powerful support for postnatal mothers [20]. A systematic review of eight qualitative studies [10] confirmed that limited time, poor staffing levels and conflicting policies are barriers to providing effective midwifery education in some contexts, which can impact on the mother’s experience while in hospital. Although a small sample, findings from both mothers and staff in our study suggest encouraging dedicated time for mothers to attend breastfeeding classes with other mothers was beneficial.

Interview findings also illuminated the importance of paying attention to small things even though time pressures were paramount. For example, saying ‘goodbye’ and ‘good luck’ to parents on discharge was valued. For many parents, taking their baby home is a time of trepidation and excitement and having staff validate these feelings is important to leaving the hospital feeling empowered and capable. A meta-synthesis by Brunstad et al. [21] reported that, for fathers, the influence of midwife interaction and communication was crucial to building confidence prior to being discharged from hospital. For mothers, the midwives’ ability to empower women is also critical as it influences the mother’s general wellbeing and comfort, particularly during the first weeks following birth. Wiklund et al. [2] report that new mothers need confirmation and positive reinforcement that they are doing well, and valued being able to discuss issues with their midwife or health professional. More specifically, their systematic review of ten studies on new parents’ experience of postnatal information delivery also confirmed that continuity of care and information delivery was appreciated, as were other small details such as knowing the name of their midwife or health professional [2].

Co-design methods gather staff and patient perspectives with the goal of improving healthcare service delivery and patient outcomes [16]. Identifying ‘touchpoints’ is important to the co-design approach because these are identified areas for improvement, highlighted by staff, consumers and patients [14]. Within our study, two main touchpoints were identified relating to timing of information delivery and the number of personnel involved in delivering information, which led to inefficiencies, feelings of being overwhelmed and mixed messages. Although both staff and patients reported positive and negative aspects, it was clear that time pressures and staffing levels influenced the overall information delivery experience. As an integral part of the co-design methodology, service improvement or redesign is a goal [16]; however, we were faced with challenges around timing and resource availability, which would need to be addressed prior to implementation of specific improvement activities.

### Limitations

Barriers to effective implementation of co-design studies are common [16,22]. During the conduct of our study, challenges were experienced that affected fidelity to co-design methods. The study was not externally funded, and we experienced some resource issues, which delayed recruitment and data collection. Our sample size was small because some mothers were reluctant to return to the hospital for the video interviews due to traffic and parking issues; others did not respond to our recruitment strategies for unknown reasons. However, as this study aimed to confirm feasibility for a larger study, we feel that the sample was sufficient for that aim. In their rapid review of research co-design in healthcare, Slattery et al. [23] highlight challenges with terminology, reporting and evaluation of such methods, in addition to issues of time and resources needed to successfully complete these kinds of studies.

Performing ward observations required a degree of empathy and tact with other staff, in addition to managerial support, to ensure ward staff were amenable, did not feel threatened and were not interrupted or distracted from their usual care delivery. Managerial support has been identified as an essential facilitator for this phase of co-design studies [16] and our experience confirmed this. Due to organizational changes, resource limitations and staff turnover, we experienced delays between data collection and the co-design phase. Limiting this gap is one of the recommendations proposed by Green et al. [16] for using EBCD methods, and future projects should take this into consideration, as these challenges influenced the ability to have the feedback events [14] that were initially planned. We did attempt to follow-up with participants via email but did not receive replies, which again may be partly influenced by the time delay. It should also be noted that since conduct of this study and taking into consideration the impact of the COVID-19 pandemic, the organization has commenced use of an online information resource for parents to augment the information delivery process.

## 5. Conclusions

Our study identified two areas of information delivery that were important to both parents, midwives, and staff within our hospital maternity setting, namely, timing of information delivery and the number of staff involved in the patient journey in such a short time, were key findings. We have also identified that managerial support and resources are essential for conducting larger co-design studies in busy hospital settings. Other methods for capturing patient experience may be explored to more appropriately address staff and parent needs in this context.

## Figures and Tables

**Table 1:** Survey responses to discharge information provided.

Item	Strongly Agree *n* (%)	Agree *n* (%)	Neither Agree Nor Disagree *n* (%)	Disagree *n* (%)	Strongly Disagree *n* (%)
The midwives understood what I needed to know regarding my own care and the care of my baby.	21 (67.7)	5 (16.1)	4 (12.9)	1 (3.2)	0
When providing education, the midwives encouraged me to look after myself as well my baby.	22 (71.0)	5 (16.1)	4 (12.9)	0	0
When I did ask a midwife a question it was answered in an individualized way. *	19 (61.3)	4 (12.9)	5 (16.1)	2 (6.5)	0
The midwife provided the rationale or reasons to why they have provided their advice.	15 (48.4)	8 (25.8)	7 (22.6)	0	1 (3.2)
The midwife asked me what I wanted to know.	14 (45.2)	9 (29.0)	6 (19.4)	2 (6.5)	0
The discharge information I received in the hospital will be useful to me at home. *	20 (64.5)	9 (29.0)	0	1 (3.2)	0
The topics covered in my discharge information were of particular interest to me. *	17 (54.8)	9 (29.0)	3 (9.7)	1 (3.2)	0
The midwives did a good job providing me with information to look after myself and my baby. *	21 (67.7)	6 (19.4)	2 (6.5)	1 (3.2)	0
Overall, I was satisfied with my discharge information. *	19 (61.3)	8 (25.8)	2 (6.5)	0	1 (3.2)

* Missing data: *n* = 1 (3.2%).

**Table 2:** Content of information delivered during ward observations.

Information Topic	Observations * n (%)
Breast-feeding	3 (75)
Sleep/settling practices	1 (25)
Infant interaction	3 (75)
Jaundice	2 (50)
Baby cares	3 (75)
Safe sleeping/SIDS	2 (50)
Adjusting to the new role	1 (25)
Postnatal depression/baby blues	1 (25)
Mothers’ self-care	1 (25)
Family planning	1 (25)
Baby massage	0
External support after discharge	4 (100)
Hospital services after discharge	1 (25)
Specific baby screening tests	2 (50)
Length of stay	0

* Refers to number of times information on the topic was observed as being delivered.

## Data Availability

Data is available through contacting the corresponding author.

## Appendix A

**Table A1:** Survey questions (example).

Please Tick a Box to Indicate Your Level of Agreement with Each Statement	Strongly Disagree				Strongly Agree
	1	2	3	4	5
The midwives understood what I needed to know regarding my own care and the care of my baby					
When providing education, the midwives encouraged me to look after myself as well my baby					
When I did ask a midwife a question, it was answered in an individualised way					
The midwife provided the rationale or reasons to why they have provided their advice					
The midwife asked me what I wanted to know					
The discharge information I received in the hospital will be useful to me at home					
The topics covered in my discharge information were of particular interest to me					
The midwives did a good job providing me with information to look after myself and my baby					
Overall, I was satisfied with my discharge information					

## Appendix B

**Table A2:** Observation record (example).

Observation start time:				Observation completion time:	
**Participants present** (please circle)					
Mother			Partner	Midwife	Allied health
Medical staff			Other		
**Topics covered in observation** (please circle)					
Breastfeeding/feeding			Sleep & settling	Infant interaction	Jaundice
Infant care e.g., bathing, nappies etc			Safe sleeping/SIDS	Adjusting to the new role	Postnatal depression/Baby Blues
Mothers’ self-care			Contraception/family planning	Baby massage	External support when home e.g., child health clinics, GP, 13Health
Services e.g., allied health clinics, parenting centre, home visiting			Specific tests e.g., hearing, NNST	Length of stay	
**General observations** (please circle)					
Staff asks parents if they have any questions		No	Yes	Unsure/Don’t know	
Spontaneous questioning by parent to staff member		Not at all	Sometimes	Frequently	Unsure/Don’t know
Staff able to answer parents’ questions		Not at all	Sometimes	Frequently	Unsure/Don’t know
Asks parents what they would like to know		No	Yes	Unsure/Don’t know	
Informs parents where they can get further information		No	Yes	Unsure/Don’t know	
Staff provides parents with written information		No	Yes	Unsure/Don’t know	
How did staff provide written information		Left information without explanation	Left information given with minimal explanation	Went through written information with parents	Not sure/Not applicable
Where did the parent place the information during interaction		Held in hands	Placed on surface	Placed in drawer	Not sure/Not applicable
Time	Participants	Description of event		Comments	
